# Ozone and Daily Mortality in Shanghai, China

**DOI:** 10.1289/ehp.9014

**Published:** 2006-05-18

**Authors:** Yunhui Zhang, Wei Huang, Stephanie J. London, Guixiang Song, Guohai Chen, Lili Jiang, Naiqing Zhao, Bingheng Chen, Haidong Kan

**Affiliations:** 1 Department of Environmental Health, School of Public Health, Fudan University, Shanghai, China; 2 Health Effects Institute, Boston, Massachusetts, USA; 3 Epidemiology Branch, National Institute of Environmental Health Sciences, National Institutes of Health, Department of Health and Human Services, Research Triangle Park, North Carolina, USA; 4 Shanghai Center of Disease Control and Prevention, Shanghai, China; 5 Shanghai Environmental Monitoring Center, Shanghai, China; 6 Department of Health Statistics, School of Public Health, Fudan University, Shanghai, China

**Keywords:** air pollution, mortality, ozone, time-series studies

## Abstract

**Background:**

Controversy remains regarding the relationship between ambient ozone and mortality worldwide. In mainland China, the largest developing country, there has been no prior study investigating the acute effect of O_3_ on death risk. Given the changes in types of air pollution from conventional coal combustion to the mixed coal combustion/motor vehicle emissions in China’s large cities, it is worthwhile to investigate the acute effect of O_3_ on mortality outcomes in the country.

**Objectives:**

We conducted a time-series study to investigate the relation between O_3_ and daily mortality in Shanghai using 4 years of daily data (2001–2004).

**Methods:**

We used the generalized additive model with penalized splines to analyze mortality, O_3_ pollution, and covariate data in warm and cold seasons. We considered daily counts of all-cause mortality and several cause-specific subcategories (respiratory and cardiovascular). We also examined these associations among several subpopulations based on age and sex.

**Results:**

O_3_ was significantly associated with total and cardiovascular mortality in the cold season but not in the warm season. In the whole-year analysis, an increase of 10 μg/m^3^ of 2-day average (lag01) O_3_ corresponds to 0.45% [95% confidence interval (CI), 0.16–0.73%], 0.53% (95% CI, 0.10–0.96%), and 0.35% (95% CI, −0.40 to 1.09%) increase of total nonaccidental, cardiovascular, and respiratory mortality, respectively. In the cold season, the estimates increased to 1.38% (95% CI, 0.68–2.07%), 1.53% (95% CI, 0.54–2.52%), and 0.95% (95% CI, −0.71 to 2.60%), respectively. In the warm season, we did not observe significant associations for both total and cause-specific mortality. The results were generally insensitive to model specifications such as lag structure of O_3_ concentrations and degree of freedom for time trend. Multipollutant models indicate that the effect of O_3_ was not confounded by particulate matter ≤ 10 μm in diameter (PM_10_) or by sulfur dioxide; however, after adding nitrogen dioxide into the model, the association of O_3_ with total and cardiovascular mortality became statistically insignificant.

**Conclusions:**

O_3_ pollution has stronger health effects in the cold than in the warm season in Shanghai. Our analyses also strengthen the rationale for further limiting levels of O_3_ pollution in outdoor air in the city.

Short-term exposure to outdoor air pollution has been linked to adverse health effects, including increased mortality, increased rates of hospital admissions and emergency department visits, exacerbation of chronic respiratory conditions (e.g., asthma), and decreased lung function (American Thoracic Society 1996; Dockery and Pope 1994). Most of these studies were conducted in the developed countries, and only a small number of studies have been conducted in Asia (Health Effects Institute 2004). In mainland China, the largest developing country, the relation between outdoor air pollution and daily mortality has been investigated in several large cities, including Beijing (Xu et al. 1994), Shenyang (Xu et al. 2000), Chongqin (Venners et al. 2003), and Shanghai (Kan and Chen 2003a, 2003b). These studies basically followed the commonly used time-series and case-crossover approaches, and their results were in accordance with those reported from Western Europe and the United States, where most epidemiologic studies were conducted.

However, there are still some key scientific issues to be addressed regarding the health effects of outdoor air pollution in China. For example, although ozone is recognized as an air pollutant that could increase death risk (Bates 2005; Bell et al. 2004, 2005; Gryparis et al. 2004; Ito et al. 2005; Levy et al. 2005; Schwartz 2005), no study has been conducted in mainland China to assess the acute effect of O_3_. Moreover, the previous findings of the effects of O_3_ on death risk have been inconsistent (Anderson et al. 2001; Borja-Aburto et al. 1997; Goldberg et al. 2001; Hoek et al. 1997; Hong et al. 1999, 2002; Ito and Thurston 1996; Loomis et al. 1999; Prescott et al. 1998); therefore, in recent regulatory impact analyses of air pollution control measures [U.S. Environmental Protection Agency (EPA) 1999a, 1999b, 2003], the U.S. EPA excluded the O_3_–mortality relationship from primary benefits estimates, stating that the epidemiologic literature was too uncertain to infer causality and provide reasonable quantitative estimates. Also, much is unknown about the synergistic effects of O_3_ and the complex mix of pollutants found in the ambient air.

China has one of the world’s worst levels of ambient air pollution. Coal has been the major source of energy in the country, constituting about 75% of all energy sources. Consequently, air pollution in China predominantly consists of coal smoke, with suspended particulate matter (PM) and sulfur dioxide as the principal air pollutants. However, with the rapid increase in the number of motor vehicles in recent years, air pollution in China’s large cities has gradually changed from the conventional coal combustion type to mixed coal combustion/motor vehicle emissions (Chen et al. 2004). Given the relatively high levels of copollutants (PM, SO_2_, and nitrogen dioxide) and change of air pollution type in China’s cities, it is worthwhile to investigate the independent effect of O_3_ on mortality outcomes in the country. Moreover, in setting air pollution control policy from a public health viewpoint, it is important to identify the health effects of air pollution from local data.

In the present study, we conducted a time-series analysis to evaluate the association between mortality outcomes (both total and cause specific) and O_3_ exposure in metropolitan Shanghai using 4 years of daily data (2001–2004).

## Materials and Methods

### Data

Daily mortality data (excluding accidents and injuries) of residents living in the nine urban districts of Shanghai from 1 January 2001 to 31 December 2004 were collected from the database of the Shanghai Municipal Center of Disease Control and Prevention. The causes of death for 2001 and 2002–2004 were coded according to the *International Classification of Diseases*, Ninth Revision [ICD-9; World Health Organization (WHO) 1977] and Tenth Revision [ICD-10 (WHO 1994)], respectively. The mortality data were classified into deaths from all causes (ICD-9 codes < 800; ICD-10 codes A00–R99), cardiovascular diseases (ICD-9 codes 390–459; ICD-10 codes I00–I99) (including subcategories such as stroke and heart diseases), and respiratory diseases (ICD-9 codes 460–519; ICD-10 codes J00–J98) [including subcategories such as chronic obstructive pulmonary disease (COPD) and acute respiratory infection]. The data were also classified by sex and age (0–4, 5–44, 45–64, ≥ 65 years) for all-cause deaths.

Daily air pollution data in 2001–2004, including O_3_, PM ≤ 10 μm in diameter (PM_10_), SO_2_, and NO_2_, were collected by the Shanghai Environmental Monitoring Center. The daily concentrations for each pollutant were averaged from the available monitoring results of six fixed-site stations under China National Quality Control located in the urban areas of Shanghai. We collected the 24-hr average concentrations for PM_10_, SO_2_, and NO_2_ and 8-hr (1000 hr to 1800 hr) average concentration for O_3_. We used the 8-hr average because it is the average time recommended by the World Health Organization (WHO) for reflecting the most health-relevant exposure to O_3_ (WHO 2000). Calculation of 24-hr average concentration of PM_10_, SO_2_, and NO_2_ required having at least 75% of the 1-hr values on that particular day. For the 8-hr average of O_3_, at least six hourly values from 1000 hr to 1800 hr had to be available. If a station had > 25% of the values missing for the whole period of analysis, the entire station was excluded from the analysis.

To allow adjustment for the effect of weather on mortality, daily (minimal, maximal, and average) temperature and humidity data were collected by the Shanghai Meteorological Bureau. The weather data were measured at a fix-site station located in Xuhui District of Shanghai.

All the mortality, pollutant, and meteorological data were validated by an independent auditing team assigned by the Health Effects Institute.

### Statistical methods

We used the generalized additive model (GAM) with penalized splines to analyze the mortality, O_3_ pollution, and covariate data from 2001 to 2004 in Shanghai. Because counts of daily mortality data typically follow a Poisson distribution, the core analysis was a GAM with log link and Poisson error that accounted for smooth fluctuations in daily mortality.

We first built the basic models for various mortality outcomes that did not include the air pollution variables. We incorporated smoothed spline functions of time and weather conditions, which can accommodate nonlinear and nonmonotonic patterns between mortality and time/weather conditions, offering a flexible modeling tool (Hastie and Tibshirani 1990). According to previous literature (Bell et al. 2004; Samet et al. 2000a, 2000b), 6 or 8 degrees of freedom (df) per year of data for time trend and 3 or 4 df (whole period of study) for temperature and relative humidity were tested. This number of degrees of freedom has been found to control well for seasonal patterns in mortality and to reduce, and often eliminate, autocorrelation. If there was over-dispersion in the variance, we used the partial autocorrelation function (PACF) to guide the selection of degrees of freedom until PACF of the residuals was < 0.1 for the first 2 lag days (independent of the associated *p*-values). In this way, we determined the optimal degree-of-freedom values per year for various causes of death in Shanghai. Other covariates, such as day of the week (DOW), were also included in the basic models. Residuals of each model were examined to check whether there were discernible patterns and autocorrelation by means of residual plots and PACF plots, respectively.

After the establishment of basic models, we introduced the pollutant variables into the models and analyzed their effects on mortality outcomes. Generalized cross-validation scores were used to compare the relative quality of the mortality predictions across these non-nested models and how well the models fit the data (Golub et al. 1979; Hastie and Tibshirani 1990).

Briefly, we fit the following log-linear GAM to obtain the estimated pollution log-relative rate β in Shanghai:


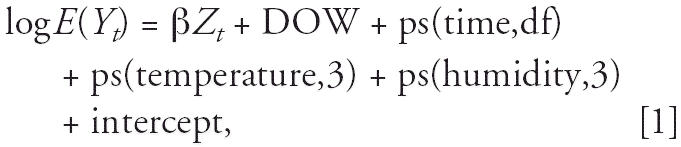


where *E*(*Y_t_*) is the expected number of deaths at day *t*; β represents the log-relative rate of mortality associated with a unit increase of air pollutants (O_3_ and copollutants); *Z_t_* indicates the pollutant concentrations at day *t*; DOW is day of the week effect; ps(time,df) is the penalized spline function of calendar time; and ps(temperature/humidity,3) is the penalized spline function for temperature/humidity with 3 df.

In a previous study, Bell et al. (2004) reported that single-day lag models under-estimate the cumulative effect of O_3_ on mortality because they take into account only 1 day’s O_3_ exposure. Therefore, in our primary analysis, we used the moving average of current and previous day concentrations of O_3_ (lag01). As a sensitivity analysis, we also examined the effect of O_3_ with different lag structures including both single-day lag and multiday lag [lag01 and the 5-day moving average of O_3_ concentrations for the previous 4-days (lag04)]. We examined the separate effect of O_3_ in the warm season (April–September) and the cold season (the remaining months). In addition, given that it is not easy to determine the optimal values of degrees of freedom for time trend in the basic models whether from statistical or biological plausibility perspectives, we did the sensitivity analysis to test the impact of degree of freedom selection on the regression results. We also fitted models with a different combination of pollutants (up to two pollutants per model) to assess the stability of O_3_’s effect.

All analyses were conducted using R 2.1.1 using the MGCV package (R Development Core Team 2006). The results are presented as the percent change in daily mortality per 10μg/m^3^ increase of O_3_, which is simply the β-coefficient from the Poisson regression × 1,000.

## Results

From 2001 to 2004, a total of 173,911 deaths (91,314 males and 82,597 females) were recorded in the study population. The four different age groups (0–4, 5–44, 45–64, and ≥ 65 years) accounted for 0.3, 3.2, 13.0, and 83.5% of the total number of deaths, respectively. On average, there were 119.0 deaths/day, among which 44.2 persons died from cardiovascular diseases and 14.3 died from respiratory diseases (Table 1). Cardiopulmonary disease accounted for 49.1% of the total nonaccidental deaths for the urban residents in Shanghai.

In our research period, the minimal, mean, and maximal daily 8-hr average concentrations of O_3_ were 5.3, 63.3, and 251.3 μg/m^3^, respectively (Table 1). For the cold and warm seasons, the mean 8-hr average O_3_ concentrations were 48.3 and 78.4 μg/m^3^, respectively.

The 8-hr average concentrations of O_3_ were weakly correlated with daily concentrations of PM_10_, SO_2_, and NO_2_ and moderately correlated with mean temperature level (Table 2). PM_10_, SO_2_, and NO_2_ had relatively higher correlation coefficients with each other.

Table 3 summarizes the effect of O_3_ on daily mortality stratified by cause of death and season in the single-pollutant models. O_3_ was significantly associated with total and cardiovascular mortality in the cold season but not in the warm season. In the whole-year analysis, an increase of 10 μg/m^3^ of 2-day average (lag01) O_3_ corresponds to 0.45% [95% confidence interval (CI), 0.16–0.73%], 0.53% (95% CI, 0.10–0.96%), and 0.35% (95% CI, −0.40to 1.09%) increase of total, cardiovascular, and respiratory mortality, respectively. In the cold season, the estimates increased to 1.38% (95% CI, 0.68–2.07%), 1.53% (95% CI, 0.54–2.52%), and 0.95% (95% CI, −0.71 to 2.60%), respectively. In the warm season, we did not observe significant associations for both total and cause-specific mortality.

For total nonaccident mortality, the estimated effect varied with sex and age groups (Table 3). The observed effect of O_3_ was larger in females than in males. For people < 65 years of age, the effects were not statistically significant, whereas for older urban residents (≥ 65 years of age), the level of O_3_ concentrations was positively associated with mortality risk.

**Figure 1:**
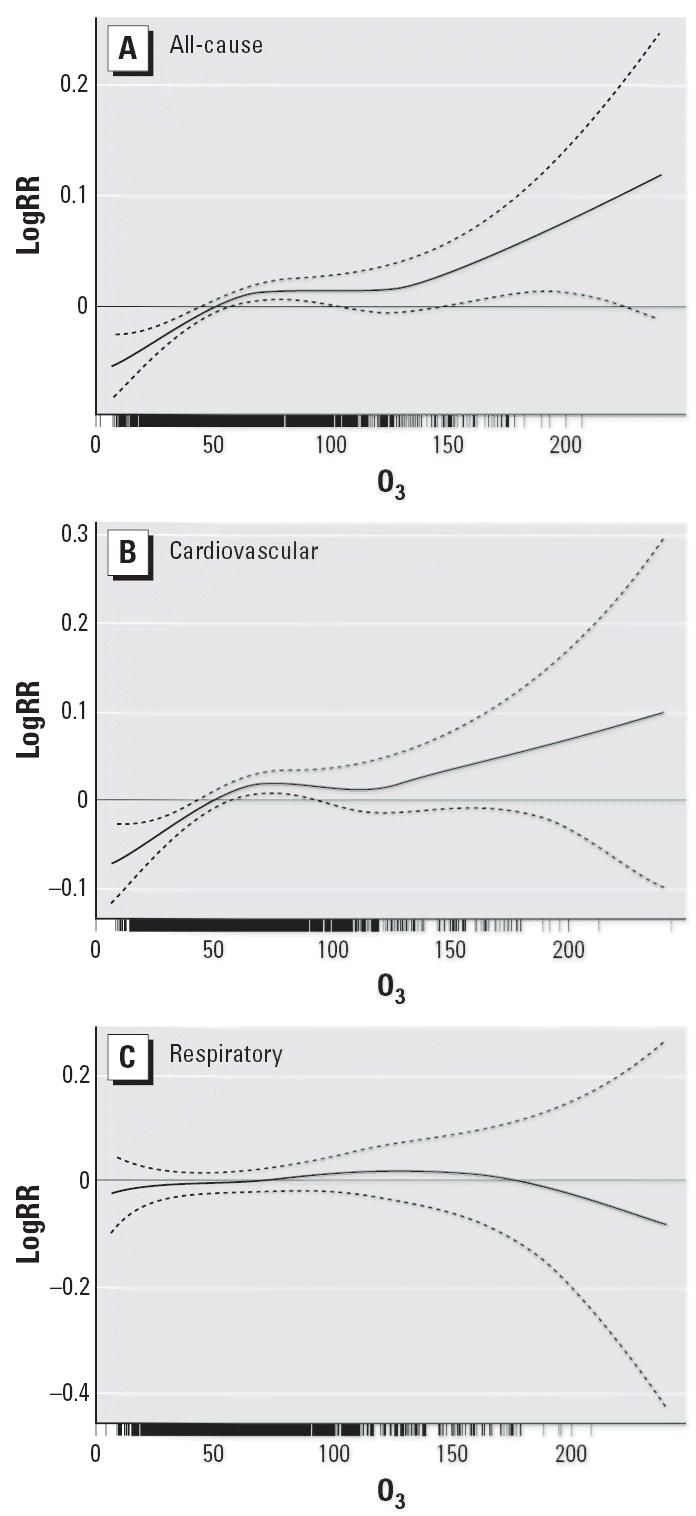
Smoothing plots of O_3_ concentration (μg/m^3^) against mortality risk (logRR, log relative risk; df = 4). (*A*) All-cause mortality. (*B*) Cardiovascular mortality. (*C*) Respiratory mortality. The solid line indicates the estimated mean percentage of change in daily mortality, and the dotted lines represent twice the pointwise SE.

For total and cardiovascular mortality, the exposure–response relationships associated with O_3_ exposure were essentially linear at concentrations < 75 μg/m^3^, although the risks were not monotonically increasing (Figure 1). The curves tended to become nonlinear and flat at higher concentrations. We did not observe any obvious threshold concentration below which O_3_ has no effect on total and cardiovascular deaths. For respiratory mortality, no clear relationship was observed.

**Figure 2:**
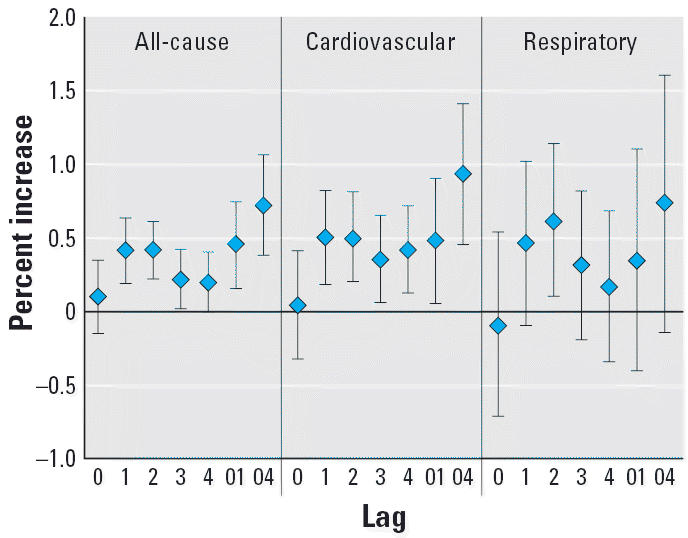
Percent increase of deaths with 10 μg/m^3^ increase of O_3_ due to all, cardiovascular, and respiratory causes in different lag days.

In our analysis, the effects of O_3_ on total and cardiovascular mortality are statistically significant for most lagged days that we examined (Figure 2). For single-day lags, O_3_ shows similar patterns for its effects on the mortality outcomes in that the risks increased from lag day 0, were maximal at lag days 1–2, and then declined. Multiday exposures (lag01 and lag04) usually have larger effects than single-day exposure. The effect of O_3_ on respiratory mortality was only significant for single-day lag 2.

**Figure 3:**
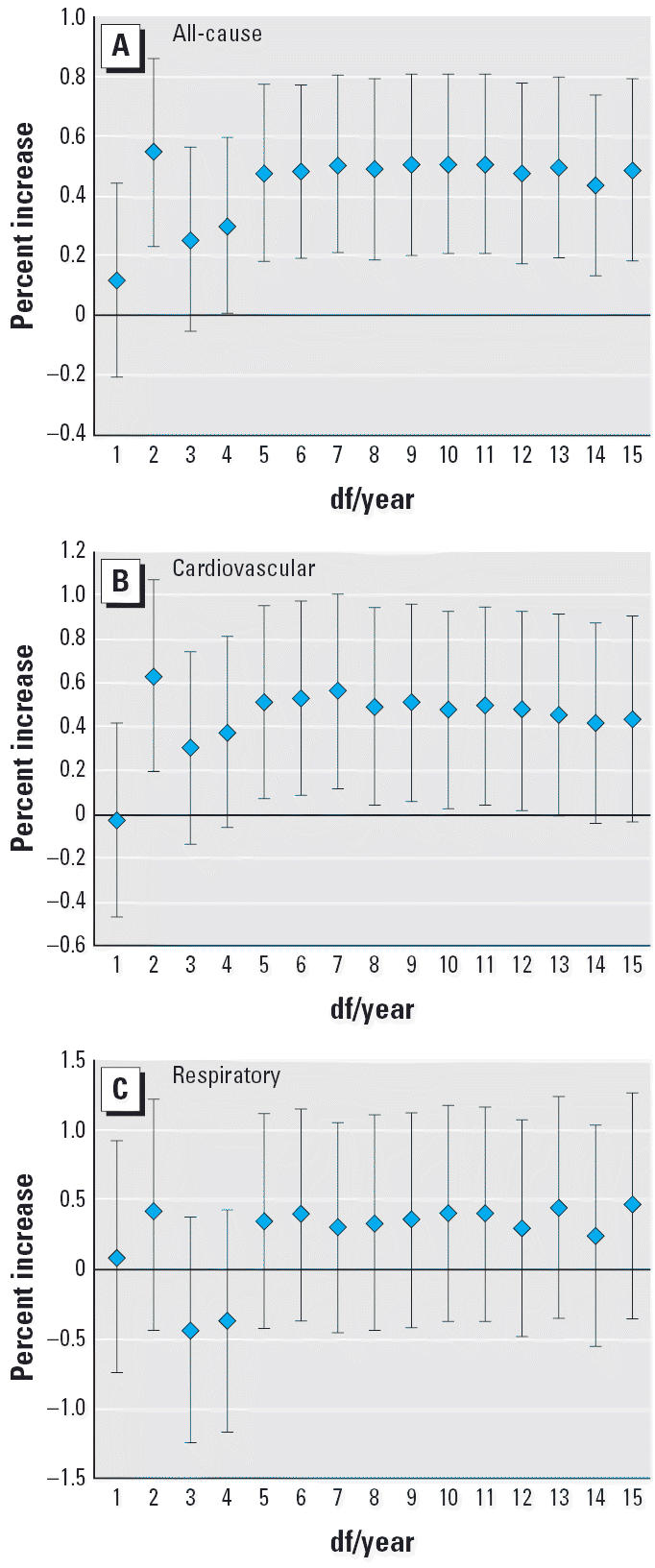
Percent increase of deaths with 10 μg/m^3^ increase of O_3_ due to all, cardiovascular, and respiratory causes classified by degrees of freedom per year. (*A*) All-cause mortality. (*B*) Cardiovascular mortality. (*C*) Respiratory mortality.

Within the range of 5–15 df, the change of degrees of freedom per year for time trend does not much affect the regression results (Figure 3), suggesting that our findings with regard to the effect of O_3_ on mortality outcomes are relatively robust.

Table 4 compares the results of the single-pollutant models and two-pollutant models. The estimated effects of O_3_ on total and cardiovascular mortality were still significant after adjustment for PM_10_ and SO_2_; however, NO_2_ was added into the regression models, the effect of O_3_ became statistically insignificant. We did not observe significant effects of O_3_ on respiratory mortality in single-pollutant or two-pollutant models.

## Discussion

Evidence gained in this study showed that the current level of O_3_ in Shanghai is associated with the death rates from all causes and from cardiovascular diseases in the cold season. To our knowledge, this is the first study to report the acute effect of O_3_ exposure on daily mortality in mainland China. Our results should contribute to the understanding of O_3_-related health effects in China and may help clarify the difference in effects and mechanisms of O_3_ between Western and Eastern populations.

Our analysis indicates an association between short-term change in O_3_ and mortality, with an estimated 0.45% increase in total mortality (95% CI, 0.16–0.73%) for a 10-μg/m^3^ increase in the 8-hr average O_3_ level at lag01 in the whole-year analysis. To compare this estimate with other studies, all estimates must be based on the same measure of O_3_ concentration, such as the 8-hr average. Most previous meta-analyses and time-series analyses used 1-hr maximal, 8-hr maximal, or daily (24-hr) average concentrations as O_3_ exposure metrics (Bell et al. 2004, 2005; Ito et al. 2005; Levy et al. 2005). A recent study of 23 European cities found a 0.34% (95% CI, 0.27–0.50) increase in daily all-cause mortality associated with a 10-μg/m^3^ increase in the average of the daily 8-hr average of the same and previous days (Gryparis et al. 2004), which is roughly comparable to our estimate. The magnitude of our estimate is also comparable to another study conducted in Hong Kong using 8-hr average O_3_ concentrations (Wong et al. 2001).

A major finding of the present study was significant effects of O_3_ on mortality outcomes only in the cold season but not in the warm season. This is consistent with two prior studies in Hong Kong (Wong et al. 1999, 2001) but in contrast to most studies in Western countries (Bell et al. 2004, 2005; Ito et al. 2005; Schwartz 2005). In Shanghai, the O_3_ level was higher in the warm season than in the cold season (mean level, 78.4 μg/m^3^ vs. 48.3 μg/m^3^), and our exposure–response relationship also reveals a flatter slope at higher concentrations (Figure 1). At higher concentrations, the risks of death could be reduced because vulnerable subjects may have died before the concentration had reached the maximum level (Wong et al. 2001). In addition, the exposure pattern may also contribute to our observation. During the warm season, Shanghai residents tend to use air conditioning more frequently because of the relatively higher temperature and humidity, thus reducing the risk of outdoor O_3_ exposure. Unstable weather conditions (heavy rain and rain storms) in the warm season also prevent the acute exposure–response relationships between O_3_ and mortality from being readily observable. In contrast, the cool season in Shanghai is drier and less variable, so people are more likely to go outdoors and open the windows. The fact that a consistently significant health effect of O_3_ was observed only in the cold season in two subtropical Asian cities (Shanghai and Hong Kong) suggests that the interaction of O_3_ exposure and weather pattern may vary by location and should be further investigated.

For total nonaccident mortality, we found a larger effect of O_3_ in females than in males. In Shanghai, male residents have a much higher smoking rate than do females (50.6% and 0.6%, respectively) (Xu 2005). A previous study (Künzli et al. 2005) suggested that the air pollution effect may be stronger in nonsmokers than in smokers. Oxidative and inflammatory effects of smoking may dominate to such an extent that the additional exposure to O_3_ may not further enhance effects along the same pathways. In addition, compared with males, females have slightly greater airway reactivity (Yunginger et al. 1992); therefore, it is possible that dose–response relations may be detected more easily in females than in males.

Our study area—nine urban districts of Shanghai—is densely populated. Within an area of 279 km^2^, there are around 7 million permanent residents and six China National Quality Control monitoring stations providing the exposure data for this study. In addition, compared with the residents in developed countries, a relatively lower proportion of Shanghai residents have access to or use air conditioning. Thus, the monitored ambient air pollution data might have been more closely associated with average population exposures in Shanghai than in other study locations of developed countries.

The limitations of our exposure assessment should also be noted. As in most previous time-series studies, we used the simply averaged monitoring results across various stations as the proxy of population exposure level to air pollution. That assignment method may raise a number of issues, given that the variance of pollutant measurements can differ from monitoring location to monitoring location and given the difference between ambient monitoring results and personal exposure level to O_3_. In addition, because O_3_ is highly reactive in indoor environments (where people spend most of their time) (Zhang and Lioy 1994), ambient O_3_ concentrations tend to be higher than personal O_3_ exposures (Avol et al. 1998). These influences challenge the accuracy of our exposure assessment and the following time-series analysis. The resulting measurement error may have substantial implications for interpreting the time-series air pollution studies (Zeger et al. 2000), although a study has suggested that this measurement error would generally tend to bias estimates downward (Samet et al. 2000b). In the future, we hope to develop an algorithm that fits the local characteristics and can be used to estimate the aggregate population exposure level to various pollutants in Shanghai.

In the single-day lag models, the estimated effects of O_3_ on mortality outcomes reached a maximum at a lag of 1–2 days. Multiday exposure (e.g., lag01 and lag04) models generally produced larger estimates compared with the single-day lag models (Figure 2). These observations are consistent with those of previous air pollution health effects reports (Bell et al. 2004; Braga et al. 2001; Zanobetti et al. 2002). This temporal pattern of effect would be anticipated for O_3_, which produces acute inflammatory responses in the lung; adaptation of this inflammatory response with several days of repeated exposure has been demonstrated (Folinsbee et al. 1994; Frank et al. 2001). Although the temporal dynamics of the underlying processes linking O_3_ exposure to increased mortality may differ from those of the inflammatory response, inflammation has been postulated as having a central role in the increased mortality and morbidity associated with O_3_ (Brook et al. 2004).

In real life, people cannot selectively inhale some air pollutants and not others. Therefore, human health effects may be the result of a complex of inhaled multipollutants, and it is very difficult to separate the effect of individual pollutants. In the present analysis, the concentration of O_3_ was weakly correlated with other pollutants. This lack of correlation and the stability of the O_3_ estimate with inclusion of PM_10_ and SO_2_ in the multipollutant models provide evidence against confounding of the effects of other pollutants. However, our estimate of O_3_ on total and cardiovascular mortality became statistically insignificant after adding NO_2_ into the model. Our observed effect of O_3_ may actually reflect the risk from the photochemical pollution mixture more generally. In addition to O_3_, atmospheric photochemistry produces several hazardous pollutants, such as peroxyacyl nitrates. O_3_ may act as a surrogate indicator for this highly complex and geographically variable mixture and is likely to be an imperfect measure of potential toxicity (Bell et al. 2004).

Our estimated effect was relatively robust to the confounding factors such as seasonality, long-term trends, temperature, and other pollutants. The results indicate a substantial health burden from O_3_ pollution. However, this value is probably an underestimate of the total mortality burden from such an increase in O_3_ because it accounts for only the short-term effects. Further, we found a relationship between mortality and O_3_ at pollution levels below the current regulatory standard. Our analysis is limited to the urban area of Shanghai, although rural communities may also experience elevated O_3_ levels, especially because of large biogenic emissions of volatile organic compounds and the movement of O_3_ and O_3_ precursors from urban regions.

Several groups within the population have been considered at increased risk from O_3_ exposure, including women, older persons, and those with underlying chronic cardiovascular diseases. Our study also confirmed the previous findings that the association between O_3_ exposure and the mortality risk of cardiovascular diseases was stronger than all-cause mortality risk. There are several possible underlying mechanisms for the possible link between O_3_ exposure and cardiovascular mortality: inflammation of pulmonary tissues, which can induce a spectrum of mediators that also may alter cardiac functions, or irritant-receptor–mediated stimulation of parasympathetic pathways (Watkinson et al. 2001). O_3_ is a potent oxidant that has been shown to produce free radicals and oxidative stress on lung cells (Ahmad et al. 2005); however, we did not observe a significant effect of O_3_ on respiratory mortality. This is consistent with the results of a recent meta-analysis from 39 time-series studies (Bell et al. 2005). The relative small number of deaths due to respiratory diseases may have limited our ability to detect small pollution association (Kinney and Ozkaynak 1991).

## Conclusion

The results presented here show an independent association between mortality outcomes and O_3_ exposure in the cold season in Shanghai. Our analyses provide evidence that the current level of O_3_ has an adverse effect on the health of the general population and strengthen the rationale for further limiting levels of O_3_ pollution in outdoor air in Shanghai.

## Figures and Tables

**Table 1:** Summary statistics of daily deaths, air pollutant concentrations, and weather conditions in Shanghai (2001–2004).

Variable	Mean ± SD	Minimum	25th percentile	Median	75th percentile	Maximum
Daily death counts						
Total (nonaccident)	119.0 ± 22.5	51.0	103.0	115.0	133.0	198.0
Cardiovascular	44.2 ± 11.0	11.0	36.0	43.0	51.0	85.0
Respiratory	14.3 ± 6.4	3.0	10.0	13.0	17.0	45.0
Air pollutant concentrations^a^						
O_3_ (μg/m^3^)	63.3 ± 36.7	5.3	37.6	56.1	82.7	251.3
PM_10_ (μg/m^3^)	102.0 ± 64.8	14.0	56.3	84.0	128.3	566.8
SO_2_ (μg/m^3^)	44.7 ± 24.2	8.4	27.5	40.0	56.2	183.3
NO_2_ (μg/m^3^)	66.6 ± 24.9	13.6	50.2	62.5	79.2	253.7
Meterologic measures						
Mean temperature (°C)	17.7 ± 8.5	−2.4	10.3	18.3	24.7	34.0
Relative humidlity (%)	72.9 ± 11.4	33.3	65.5	73.5	81.0	97.0

a Twenty-four-hour average for PM_10_, SO_2_, and NO_2_; 8-hr (1000 hr to 1800 hr) average for O_3_.

**Table 2:** Correlation coefficients between daily air pollutant concentrations and weather conditions in metropolitan Shanghai (2001–2004).^a^

	SO_2_	NO_2_	O_3_	Temperature	Relative humidity
PM_10_	0.64	0.71	0.19	−0.21	−0.37
SO_2_	1.00	0.73	0.14	−0.21	−0.52
NO_2_		1.00	0.01	−0.38	−0.27
O_3_			1.00	0.48	−0.35
Temperature				1.00	0.21

a Twenty-four-hour average for PM_10_, SO_2_, and NO_2_; 8-hr (1000 hr to 1800 hr) average for O_3_.

**Table 3:** Percent increase (95% CI) of mortality outcomes of Shanghai residents associated with a 10-μg/m^3^ increase in O_3_ concentrations in 2001–2004.^a^

Cause of death	Daily deaths (*n*)	Full year	Cold season	Warm season
All causes				
Total (nonaccident)	119.0	0.45 (0.16 to 0.73)	1.38 (0.68 to 2.07)	0.30 (−0.01 to 0.61)
Total, male	62.5	0.37 (0.01 to 0.73)	1.02 (0.17 to 1.87)	0.25 (−0.17 to 0.66)
Total, female	56.5	0.53 (0.15 to 0.90)	1.78 (0.87 to 2.68)	0.37 (−0.04 to 0.77)
Total (0–4 years)	0.3	−3.88 (−8.64 to 0.88)	−6.09 (−17.14 to 4.97)	−4.33 (−10.16 to 1.50)
Total (5–44 years)	3.7	−0.13 (−1.48 to 1.22)	0.59 (−2.73 to 3.91)	0.19 (−1.35 to 1.72)
Total (45–64 years)	15.5	0.56 (−0.11 to 1.23)	1.65 (0.03 to 3.27)	0.23 (−0.55 to 1.01)
Total (≥ 65 years)	99.6	0.46 (0.16 to 0.77)	1.38 (0.65 to 2.11)	0.33 (0.00 to 0.66)
Cardiovascular disease	44.2	0.53 (0.10 to 0.96)	1.53 (0.54 to 2.52)	0.37 (−0.12 to 0.85)
Stroke	25.5	0.79 (0.23 to 1.35)	1.74 (0.49 to 2.98)	0.57 (−0.09 to 1.22)
Heart disease	16.8	0.24 (−0.43 to 0.92)	1.16 (−0.33 to 2.66)	0.14 (−0.66 to 0.94)
Respiratory disease	14.3	0.35 (−0.40 to 1.09)	0.95 (−0.71 to 2.60)	0.14 (−0.71 to 0.99)
COPD	12.2	0.22 (−0.60 to 1.03)	0.75 (−1.05 to 2.54)	0.07 (−0.86 to 1.00)
Acute respiratory infection	1.0	1.99 (−0.55 to 4.52)	0.73 (−5.08 to 6.53)	1.93 (−1.13 to 4.99)

a Current day temperature and relative humidity (lag = 0), and 2-day moving average of O_3_ concentrations (lag01) were used in all the regression models shown in this table.

**Table 4:** Percent increase of total, cardiovascular, and respiratory mortality associated with a 10-μg/m^3^ increase of 2-day average O_3_ concentrations under single- and two-pollutant models.^a^

Cause of death	Mean percent (95% CI)
Total mortality	
Single-pollutant model	0.45 (0.16 to 0.73)
Adjusted for PM_10_	0.35 (0.06 to 0.64)
Adjusted for SO_2_	0.34 (0.05 to 0.63)
Adjusted for NO_2_	0.26 (−0.03 to 0.55)
Cardiovascular mortality	
Single-pollutant model	0.53 (0.10 to 0.96)
Adjusted for PM_10_	0.44 (0.00 to 0.88)
Adjusted for SO_2_	0.44 (0.00 to 0.87)
Adjusted for NO_2_	0.35 (−0.09 to 0.79)
Respiratory mortality	
Single-pollutant model	0.35 (−0.40 to 1.09)
Adjusted for PM_10_	0.24 (−0.51 to 1.00)
Adjusted for SO_2_	0.18 (−0.57 to 0.92)
Adjusted for NO_2_	0.07 (−0.69 to 0.82)

a Current day temperature and relative humidity (lag 0), 2-day moving average of O_3_ and copollutants (PM_10_, SO_2_, and NO_2_) concentrations (lag01) were used in all the regression models shown.

## References

[b1-ehp0114-001227] Ahmad S, Ahmad A, McConville G, Schneider BK, Allen CB, Manzer R (2005). Lung epithelial cells release ATP during ozone exposure: signaling for
cell survival. Free Radic Biol Med.

[b2-ehp0114-001227] American Thoracic Society, Committee of the Environmental and Occupational
Health Assembly (1996). Health effects of outdoor air pollution. Am J Respir Crit Care Med.

[b3-ehp0114-001227] Anderson HR, Bremner SA, Atkinson RW, Harrison RM, Walters S (2001). Particulate matter and daily mortality and hospital admissions in the West
Midlands conurbation of the United Kingdom. Occup Environ Med.

[b4-ehp0114-001227] Avol EL, Navidi WC, Colome SD (1998). Modeling ozone levels in and around Southern California homes. Environ Sci Technol.

[b5-ehp0114-001227] Bates D (2005). Ambient ozone and mortality. Epidemiology.

[b6-ehp0114-001227] Bell ML, Dominici F, Samet JM (2005). A meta-analysis of time-series studies of ozone and mortality with comparison
to the national morbidity, mortality, and air pollution study. Epidemiology.

[b7-ehp0114-001227] Bell ML, McDermott A, Zeger SL, Samet JM, Dominici F (2004). Ozone and short-term mortality in 95 US urban communities, 1987–2000. JAMA.

[b8-ehp0114-001227] Borja-Aburto VH, Loomis DP, Bangdiwala SI, Shy CM, Rascon-Pacheco RA (1997). Ozone, suspended particulates, and daily mortality in Mexico City. Am J Epidemiol.

[b9-ehp0114-001227] Braga AL, Zanobetti A, Schwartz J (2001). The lag structure between particulate air pollution and respiratory and
cardiovascular deaths in 10 US cities. J Occup Environ Med.

[b10-ehp0114-001227] Brook RD, Franklin B, Cascio W, Hong Y, Howard G, Lipsett M (2004). Air pollution and cardiovascular disease: a statement for healthcare professionals
from the Expert Panel on Population and Prevention Science
of the American Heart Association. Circulation.

[b11-ehp0114-001227] Chen B, Hong C, Kan H (2004). Exposures and health outcomes from outdoor air pollutants in China. Toxicology.

[b12-ehp0114-001227] Dockery DW, Pope CA (1994). Acute respiratory effects of particulate air pollution. Annu Rev Public Health.

[b13-ehp0114-001227] Folinsbee LJ, Horstman DH, Kehrl HR, Harder S, Abdul-Salaam S, Ives PJ (1994). Respiratory responses to repeated prolonged exposure to 0.12 ppm ozone. Am J Respir Crit Care Med.

[b14-ehp0114-001227] Frank R, Liu MC, Spannhake EW, Mlynarek S, Macri K, Weinmann GG (2001). Repetitive ozone exposure of young adults: evidence of persistent small
airway dysfunction. Am J Respir Crit Care Med.

[b15-ehp0114-001227] Goldberg MS, Burnett RT, Brook J, Bailar JC, Valois MF, Vincent R (2001). Associations between daily cause-specific mortality and concentrations
of ground-level ozone in Montreal, Quebec. Am J Epidemiol.

[b16-ehp0114-001227] Golub GH, Heath M, Wahba G (1979). Generalized cross-validation as a method for choosing a good ridge parameter. Technometrics.

[b17-ehp0114-001227] Gryparis A, Forsberg B, Katsouyanni K, Analitis A, Touloumi G, Schwartz J (2004). Acute effects of ozone on mortality from the “Air Pollution and
Health: A European Approach” project. Am J Respir Crit Care Med.

[b18-ehp0114-001227] Hastie TJ Tibshirani RJ 1990. Generalized Additive Models. London:Chapman & Hall.

[b19-ehp0114-001227] Health Effects Institute 2004. Health Effects of Outdoor Air Pollution in Developing Countries of Asia: A Literature Review. Boston:Health Effects Institute.

[b20-ehp0114-001227] Hoek G, Schwartz JD, Groot B, Eilers P (1997). Effects of ambient particulate matter and ozone on daily mortality in Rotterdam, the
Netherlands. Arch Environ Health.

[b21-ehp0114-001227] Hong Y-C, Lee J-T, Kim H, Kwon H-J (2002). Air pollution: a new risk factor in ischemic stroke mortality. Stroke.

[b22-ehp0114-001227] Hong Y-C, Leem J-H, Ha E-H, Christiani DC (1999). PM_10_ exposure, gaseous pollutants, and daily mortality in Ichon, South Korea. Environ Health Perspect.

[b23-ehp0114-001227] Ito K, De Leon SF, Lippmann M (2005). Associations between ozone and daily mortality: analysis and meta-analysis. Epidemiology.

[b24-ehp0114-001227] Ito K, Thurston GD (1996). Daily PM_10_/mortality associations: an investigation of at-risk subpopulations. J Expo Anal Environ Epidemiol.

[b25-ehp0114-001227] Kan H, Chen B (2003a). Air pollution and daily mortality in Shanghai: a time series study. Arch Environ Health.

[b26-ehp0114-001227] Kan H, Chen B (2003b). A case-crossover analysis of air pollution and daily mortality in Shanghai. J Occup Health.

[b27-ehp0114-001227] Kinney PL, Ozkaynak H (1991). Associations of daily mortality and air pollution in Los Angeles County. Environ Res.

[b28-ehp0114-001227] Künzli N, Jerrett M, Mack WJ, Beckerman B, LaBree L, Gilliland F (2005). Ambient air pollution and atherosclerosis in Los Angeles. Environ Health Perspect.

[b29-ehp0114-001227] Levy JI, Chemerynski SM, Sarnat JA (2005). Ozone exposure and mortality: an empiric Bayes metaregression analysis. Epidemiology.

[b30-ehp0114-001227] Loomis D, Castillejos M, Gold DR, McDonnell W, Borja-Aburto VH (1999). Air pollution and infant mortality in Mexico City. Epidemiology.

[b31-ehp0114-001227] Prescott GJ, Cohen GR, Elton RA, Fowkes FG, Agius RM (1998). Urban air pollution and cardiopulmonary ill health. Occup Environ Med.

[b32-ehp0114-001227] R Development Core Team 2006. R: A Language and Environment for Statistical Computing, version 2.1.1. Vienna:R Foundation for Statistical Computing.

[b33-ehp0114-001227] Samet JM, Dominici F, Curriero FC, Coursac I, Zeger SL (2000a). Fine particulate air pollution and mortality in 20 U.S. cities, 1987–1994. N Engl J Med.

[b34-ehp0114-001227] Samet JM, Dominici F, Zeger SL, Schwartz J, Dockery DW (2000b). The National Morbidity, Mortality, and Air Pollution Study. Part I: Methods
and methodologic issues. Res Rep Health Eff Inst.

[b35-ehp0114-001227] Schwartz J (2005). How sensitive is the association between ozone and daily deaths to control
for temperature?. Am J Respir Crit Care Med.

[b36-ehp0114-001227] U.S. EPA 1999a. The Benefits and Costs of the Clean Air Act: 1990 to 2010. Washington, DC:U.S. Environmental Protection Agency, Office of Air and Radiation.

[b37-ehp0114-001227] U.S. EPA 1999b. Regulatory Impact Analysis—Control of Air Pollution from New Motor Vehicles: Tier 2 Motor Vehicle Emissions Standards and Gasoline Sulfur Control Requirements. Washington, DC:U.S. Environmental Protection Agency, Office of Air and Radiation.

[b38-ehp0114-001227] U.S. EPA 2003. Draft Regulatory Impact Analysis: Control of Emissions from Nonroad Diesel Engines. Washington, DC:U.S. Environmental Protection Agency, Assessment and Standards Division, Office of Transportation and Air Quality.

[b39-ehp0114-001227] Venners SA, Wang B, Xu Z, Schlatter Y, Wang L, Xu X (2003). Particulate matter, sulfur dioxide, and daily mortality in Chongqing, China. Environ Health Perspect.

[b40-ehp0114-001227] Watkinson WP, Campen MJ, Nolan JP, Costa DL (2001). Cardiovascular and systemic responses to inhaled pollutants in rodents: effects
of ozone and particulate matter. Environ Health Perspect.

[b41-ehp0114-001227] WHO 1977. International Classification of Diseases, Ninth Revision. Geneva:World Health Organization.

[b42-ehp0114-001227] WHO 1994. International Classification of Diseases, Tenth Revision. Geneva:World Health Organization.

[b43-ehp0114-001227] WHO 2000. Air Quality Guideline for Europe. WHO Regional Publication, European Series, No. 91. Copenhagen:World Health Organization. 11372513

[b44-ehp0114-001227] Wong CM, Ma S, Hedley AJ, Lam TH (1999). Does ozone have any effect on daily hospital admissions for circulatory
diseases?. J Epidemiol Community Health.

[b45-ehp0114-001227] Wong CM, Ma S, Hedley AJ, Lam TH (2001). Effect of air pollution on daily mortality in Hong Kong. Environ Health Perspect.

[b46-ehp0114-001227] Xu X, Gao J, Dockery DW, Chen Y (1994). Air pollution and daily mortality in residential areas of Beijing, China. Arch Environ Health.

[b47-ehp0114-001227] Xu Z (2005). Effect evaluation on smoking control plan for one year in Shanghai-China/WHO
smoking control capability construction cooperation items [in
Chinese]. Chin J Health Educ.

[b48-ehp0114-001227] Xu Z, Yu D, Jing L, Xu X (2000). Air pollution and daily mortality in Shenyang, China. Arch Environ Health.

[b49-ehp0114-001227] Yunginger JW, Reed CE, O’Connell EJ, Melton LJ, O’Fallon WM, Silverstein MD (1992). A community-based study of the epidemiology of asthma. Incidence rates, 1964–1983. Am Rev Respir Dis.

[b50-ehp0114-001227] Zanobetti A, Schwartz J, Samoli E, Gryparis A, Touloumi G, Atkinson R (2002). The temporal pattern of mortality responses to air pollution: a multicity
assessment of mortality displacement. Epidemiology.

[b51-ehp0114-001227] Zeger SL, Thomas D, Dominici F, Samet JM, Schwartz J, Dockery D (2000). Exposure measurement error in time-series studies of air pollution: concepts
and consequences. Environ Health Perspect.

[b52-ehp0114-001227] Zhang J, Lioy PJ (1994). Ozone in residential air: concentrations, I/O ratios, indoor chemistry, and
exposures. Indoor Air.

